# Treatment of paroxysmal and persistent atrial fibrillation with pulsed field ablation

**DOI:** 10.1093/europace/euag194

**Published:** 2026-10-21

**Authors:** Serge Boveda, Kyoung-Ryul Julian Chun, Pierre Jais, Nicolas Johner, Claire A Martin, Rami Daya, Andrea Sarkozy

**Affiliations:** Heart Rhythm Management Department, Clinique Pasteur, 45 Avenue de Lombez, 31300 Toulouse, France; Klinik fur Rhythmologie, CCB am Markus Krankenhaus, Wilhelm-Epstein 4, 60431 Frankfurt, Germany; Klinik fur Rhythmologie, Universitatsklinikum Schleswig-Holstein (UKSH), Ratzeburger Allee 160, 23548 Lubeck, Germany; Hopital Cardiologique du Haut-Leveque, CHU de Bordeaux, Avenue de Magellan, 33600 Pessac, France; IHU LIRYC, IHU LIRYC Bordeaux University, Av. du Haut Leveque, 33600 Pessac, France; Cardiology Department, American Hospital of Paris, 84 Boulevard de la Saussaye, 92200 Neuilly-Sur-Seine, France; Hopital Cardiologique du Haut-Leveque, CHU de Bordeaux, Avenue de Magellan, 33600 Pessac, France; IHU LIRYC, IHU LIRYC Bordeaux University, Av. du Haut Leveque, 33600 Pessac, France; Department of Cardiology, Royal Papworth Hospital, Cambridge Biomedical Campus, Cambridge CB2 0AY, United Kingdom; Department of Clinical Medicine, University of Cambridge, Trinity Lane, Cambridge CB2 1TN, United Kingdom; Rhythm Management Medical Affairs, Boston Scientific, Masaref Street, 13-6045 Beirut, Lebanon; Heart Rhythm Management Centre, Universitair Ziekenhuis Brussel, Vrije Universiteit Brussel, 1090 Jette, Brussel, Belgium

**Keywords:** Pulsed field ablation, Atrial fibrillation, Paroxysmal atrial fibrillation, Persistent atrial fibrillation, Procedural workflow, Safety, Procedural efficacy, Mid and long term efficacy, Clinical evidence, Measurable outcomes, Blanking period definition for PFA, PVI durability, AF burden, Patient reported measurable outcomes, Post-ablation recovery and return to activity, Patient-reported outcomes and quality of life

## Abstract

Pulsed field ablation has emerged as a novel energy source for atrial fibrillation ablation, offering preferential myocardial ablation with a favourable safety profile. Contemporary paroxysmal AF clinical studies demonstrate 12-month freedom from atrial arrhythmia recurrence in approximately 75-85% of patients, with major complication rates generally ranging from 1 to 3%. Compared with thermal ablation, PFA offers comparable efficacy and streamlined procedural workflows. In persistent AF, PVI remains the foundation of ablation, while additional adjunctive strategies are increasingly evaluated. Current evidence suggests that PFA can achieve 12-month arrhythmia-free outcomes generally ranging from approximately 60-75% in persistent AF populations. Major procedure-related complications typically occur in <3% of cases, supporting the feasibility of broader lesion sets with an acceptable safety profile. However, the incremental benefit of adjunctive ablation beyond PVI and the optimal lesion set for persistent AF remain to be assessed further Outcome assessment after PFA requires consideration of evolving endpoints. The clinical relevance of the traditional post-ablation blanking period is being re-evaluated, while continuous rhythm monitoring and AF burden are increasingly incorporated alongside conventional recurrence endpoints. Patient-reported outcomes are an important component of PFA evaluation. Compared with thermal ablation, PFA is associated with improved procedural tolerability, reduced analgesic requirements, rapid recovery, and the potential for same-day discharge. These features, together with favourable efficacy and safety outcomes, may contribute to broader adoption of PFA across both paroxysmal and persistent AF populations.

## Graphical Abstract

**Graphical Abstract:**
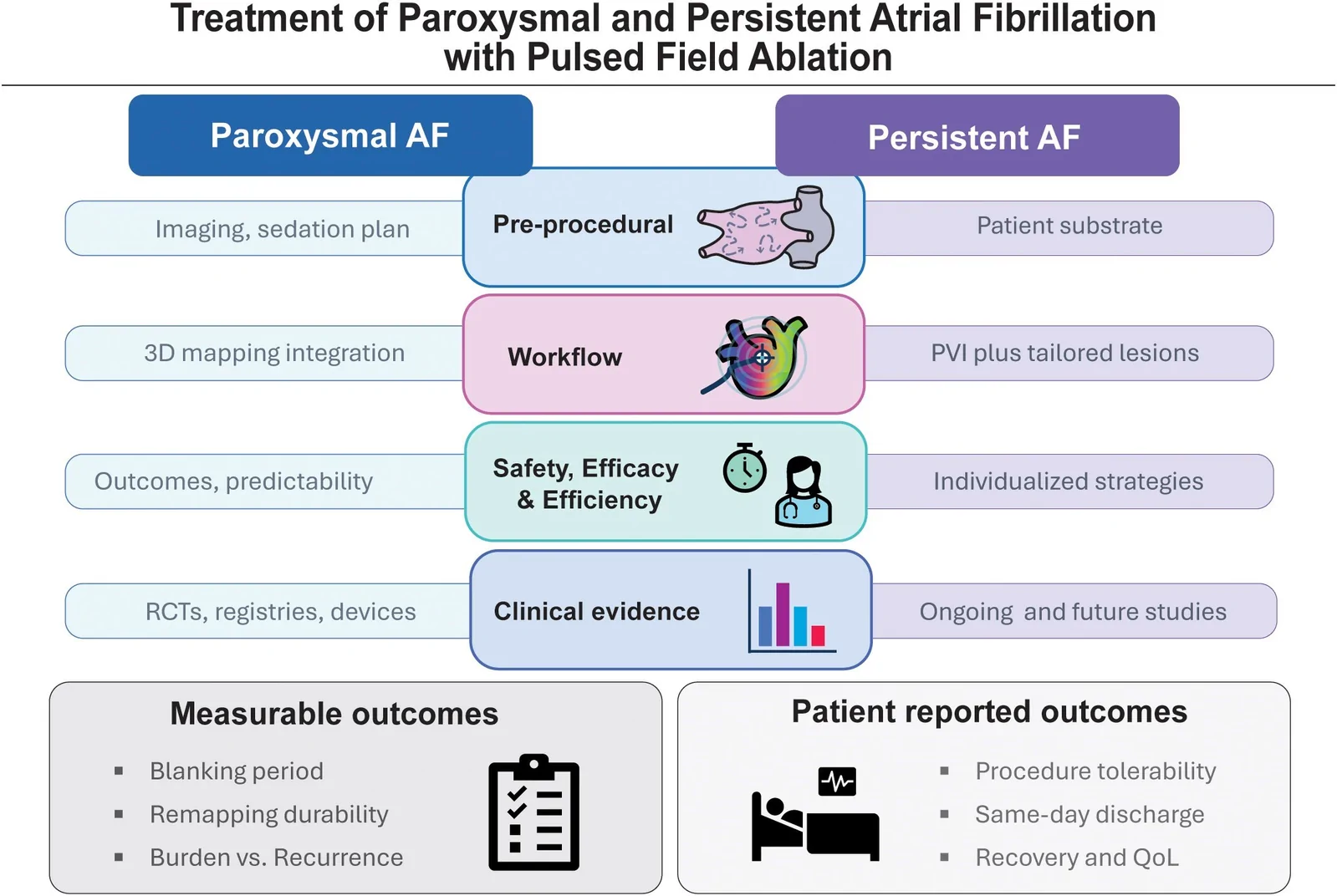

## Introduction

Pulsed field ablation (PFA) has emerged as a largely non-thermal energy modality that utilizes the principle of irreversible electroporation to achieve myocardial tissue ablation. By preferentially targeting cardiomyocytes while sparing adjacent structures such as the oesophagus and phrenic nerve and avoiding pulmonary vein stenosis, PFA offers the potential for improved safety and procedural efficiency for atrial fibrillation (AF) ablation. Early clinical experience and growing evidence suggest that PFA may overcome several limitations of conventional ablation technologies, with shorter procedure times, a favourable safety profile, and promising efficacy outcomes.

As PFA technology rapidly evolves, its application across different AF populations—including paroxysmal AF (PAF) and persistent AF (PersAF)—is being actively investigated. Key considerations include patient selection, procedural workflows, procedural efficacy, safety and efficiency, integration with advanced three-dimensional mapping systems, and durability of lesion formation. In parallel, there is increasing interest in measurable clinical outcomes of AF ablation, ranging from arrhythmia recurrence and AF burden to patient-reported quality of life and recovery trajectories.

This document provides a comprehensive overview of PFA in contemporary AF management. It explores procedural strategies in PAF and PersAF, evaluates clinical and patient-centred outcomes, and highlights current evidence as well as ongoing research efforts. Together, these insights aim to inform clinical decision-making and guide future developments in the field of AF ablation.

## PFA in paroxysmal AF

### Introduction

Pulmonary vein isolation (PVI) represents the procedural cornerstone and primary endpoint of catheter ablation for PAF.^1^ Conventional radiofrequency (RF) and cryoballoon (CB) PVI technologies rely on thermal tissue injury and are therefore associated with the risk of collateral damage to adjacent structures. PFA has emerged as a predominantly non-thermal energy modality and is regarded as a potentially transformative technology for AF ablation, particularly in PAF, with rapid adoption into clinical practice.^2^ This section focuses on established devices currently used in routine clinical practice.

### Pre-procedural considerations

#### Imaging

The EHRA clinical consensus document alludes to the importance of cardiac computed tomography (CT) or magnetic resonance (MR) imaging for anatomic and substrate delineation in complex AF and ventricular tachycardia (VT) ablation procedures.^3^ In general, pre-procedural imaging remains institution- and workflow-dependent and may include no dedicated imaging, cardiac MR imaging, or CT. Its use should not differ from thermal ablation protocols. Given the availability of large-diameter, anatomy-based single-shot PVI catheters, omission of systematic pre-procedural imaging is feasible and widespread. Similar to thermal energy PVI procedures, to exclude left atrial thrombus, transoesophageal echocardiography, intra-cardiac echocardiography (ICE), or cardiac CT is recommended in patients with a CHA_2_DS_2_-VA score ≥1 who have not received continuous oral anticoagulation (OAC) for at least 3 weeks.^1^ OAC is generally managed uninterrupted or with omission of the morning dose on the day of the procedure and resumption in the evening.

#### Sedation vs. general anaesthesia

A systematic review and meta-analysis of 12 302 patients undergoing catheter ablation for AF showed that there was no significant difference in recurrence of atrial tachyarrhythmias between general anaesthesia (GA) and sedation.^4^ Similarly, a prospective registry of 1002 patients showed that conscious sedation is as safe and effective as GA in AF ablation, particularly with cryoablation. The choice of anaesthesia appears to be driven by patient characteristics and institutional factors without affecting long-term outcomes such as AF recurrence or complication rates.^5^

Skeletal muscle activation during PFA generally necessitates deep sedation or GA to ensure patient comfort and catheter stability, even though conscious sedation has been used successfully with some PFA platforms.^6^ Initial European experience has demonstrated feasibility and safety of deep sedation with both bipolar and monopolar PFA systems.^7,8^ Importantly, the sedation strategy is typically guided by national regulations and institutional practice.^9^ While GA is frequently adopted in the United States, deep sedation is commonly used in several European countries and supported by a well-trained electrophysiology team.

### Procedural workflow and 3D mapping integration for PVI

For PVI-only procedures, the introduction of single-shot technologies led to a shift from point-by-point RF ablation towards anatomy-based approaches, which was rapidly adopted in early PFA systems.^10^ After femoral venous access and transseptal puncture, contemporary anatomy-based PFA systems enable rapid PVI using multielectrode catheters delivering high-voltage pulses. Catheter positioning may be guided by fluoroscopy alone or combined with ICE or 3D mapping. Energy delivery at the PV ostium or antrum, often with catheter rotations, results in acute elimination of PV potentials. Anticholinergic agents or RSPV first isolation may mitigate vagal reflexes.^11–13^ More recent PFA platforms integrate advanced 3D mapping and/or ICE to assess catheter positioning and tissue contact, facilitating tailored strategies, including substrate-based targets beyond the PVs or the posterior LA wall. Owing to its favourable safety profile, PFA has generally reduced or obviated routine use of oesophageal temperature monitoring in many centres, although practices vary. Although some strategies support fluoroscopy-guided workflows,^14^ 3D mapping is increasingly adopted, providing anatomical visualizations, catheter navigation, lesion prediction, impedance-based contact assessment, and identification of low-voltage areas.^15,16^ While PFA-based PVI can be performed with minimal mapping, 3D mapping may facilitate lesion documentation, confirmation of isolation, and voltage assessment. In the MANIFEST-REDO study, the use of imaging or 3D mapping during *de novo* PVI was associated with greater PVI durability.^17^ However, to date, no RCT has been carried out to show improved clinical success with routine 3D mapping in PFA, supporting its role as an adjunct.^18^

### Clinical studies

Clinical evidence for PFA in PAF includes RCTs, large real-world registries, and procedural refinement studies addressing lesion durability.

#### Randomized controlled trials

RCTs available with the pentaspline catheter consistently demonstrate non-inferiority of PFA compared with thermal ablation. In ADVENT,^18^ PFA achieved comparable 12-month freedom from atrial arrhythmia recurrence vs. RF and CB ablation, with high acute PVI rates and a favourable safety profile despite limited operator experience with PFA. In single Shot Champion, experienced operators confirmed non-inferiority of single-shot PFA vs. CB ablation, with a signal towards improved efficacy.^19^ Continuous monitoring with implantable loop recorders (ILRs) showed comparable AF burden.^20^ Most recently, the BEAT PAROX-AF trial was published demonstrating comparable PVI success rates with the pentaspline PFA vs. focal RF energy (77, 2% vs. 77, 6%) in experienced centres using the CLOSE protocol (*Table 1*). Importantly, after RF ablation, PV stenosis was noted in 12 patients (*n* = 2 > 70%, *n* = 10 > 50%, 11.3%). Clinical efficacy outcomes with the pentaspline catheter in all RCTs were comparable, while procedural metrics and safety favoured PFA (*Figure 1*).

**Figure 1:**
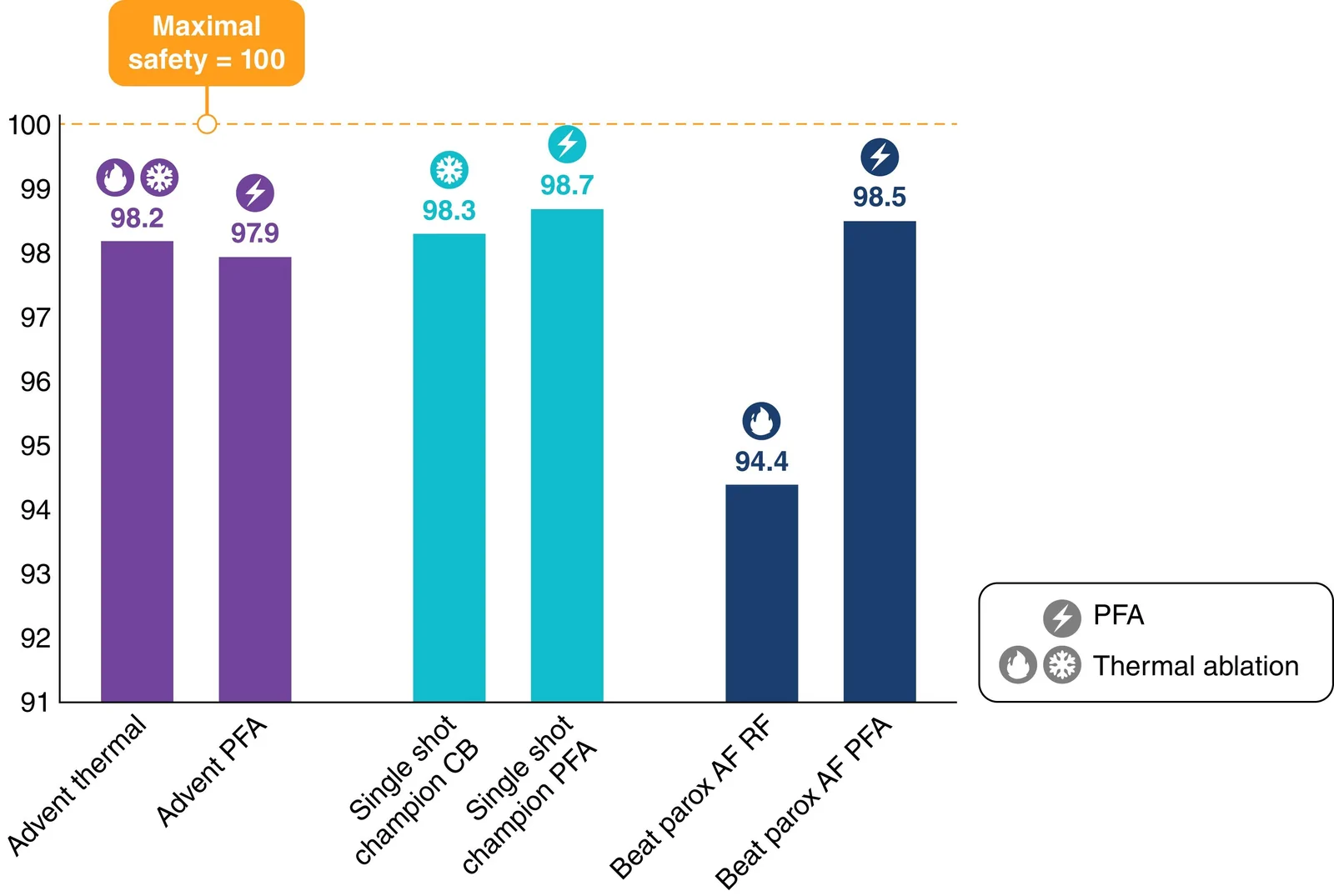
Safety profiles based on the proposed EHRA Complication Score. Based on the proposed EHRA complication score, safety indices derived from recent randomized controlled trials comparing pulsed-field ablation with thermal ablation confirm and quantitatively support the improved safety profile of PFA. Maximal safety would render a score of 100.

**Table 1:** Randomized clinical trials of pulsed field ablation vs. thermal ablation

	ADVENT (2023)	SINGLE SHOT (2025)	BEAT PAROX-AF (2026)
Comparator	RF/CB	CB	RF
Patients (n)	305	210	292
Mean Procedure time (min)	PFA 105.8 ± 29.4 Thermal 123.1 ± 42.1	PFA 54.8 ± 22.7 CB 73.2 ± 26.7	PFA 59 ± 22 RF 98 ± 33
Mean Fluoroscopy time (min)	PFA 21.1 ± 11.0 Thermal 13.9 ± 12.8	PFA 14.6 ± 7.2 CB 15.1 7.9	PFA 12 ± 6 RF 7 ± 6
Acute PVI success	>99%	100%	100%
Primary Efficacy Endpoint	Freedom from a composite of initial procedural failure to achieve pulmonary vein isolation with the use of the randomly assigned treatment method only, documented atrial tachyarrhythmia lasting 30 s or longer after the 3-month blanking period, the use of class I or III antiarrhythmic drugs or cardioversion after the 3-month blanking period, the use of amiodarone at any time, or repeat ablation at any time during the 1-year follow-up period	The first recurrence of any atrial tachyarrhythmia (atrial fibrillation, flutter, or tachycardia) lasting at least 30 s during the period from Days 91 to 365 after the ablation procedure	Single-procedure success rate after 12 months defined as the absence of ≥30 s atrial arrhythmia recurrence, class I/III antiarrhythmic drug resumption after a 2-mo blanking period, or any repeat ablation
Primary Safety Endpoint	A composite of prespecified device- and procedure-related serious adverse events within 7 days after the procedure. Atrioesophageal fistula and pulmonary vein stenosis were included as serious adverse events regardless of the timing of occurrence	A composite of cardiac tamponade leading to pericardiocentesis, persistent phrenic-nerve palsy lasting more than 24 h, serious vascular complications leading to intervention, stroke or transient ischemic attack, atrioesophageal fistula, or death within 30 days	Composite safety endpoint of at least one adverse event per patient
Monitoring method	TTM: weekly (3–12 mo) + when symptomatic ECG: 3, 6, and 12 months Holter: 72 h at 6 and 12 months	Continuous rhythm monitoring after ablation with implantable cardiac monitors	Telephone follow-up at 1 month, in-person follow-up at 2, 6, and 12 months. Weekly self-recorded single-lead ECGs with hand-held ECG (AliveCor Kardia) and symptom-driven recordings
Primary efficacy	PFA 73.3% Thermal 71.3%	PFA 62.9% CB 49.3%	PFA 77.2% RF 77.6%
Primary safety	PFA 2.1% Thermal 1.3%	PFA 0.95% CB: 1.9%	PFA 4.8% RF 7.6%

Summary of main outcome reports.

PFA, pulsed field ablation; RF, radiofrequency current; CB, cryoballoon; CC, complication class; FU, follow up.

#### Registry and real-world studies

Large multicentre registries covering different workflows extend these findings to routine practice, reporting near-universal 100% acute PVI, elimination of major thermal complications, and mid-term single-procedure arrhythmia-free survival of ∼70–>80%, depending on monitoring strategy.^21,22^

#### Durability optimization: the olive strategy

Procedural techniques can influence PVI durability. In a single centre study with the pentaspline catheter, the ‘olive’ strategy (2 PF applications in a smaller basket configuration placement into the PV ostium) seemed to improve lesion durability without compromising safety based on invasive remapping suggesting reduced PV reconnection and no increase in complexity or complications^23^ (*Figure 2*).

**Figure 2:**
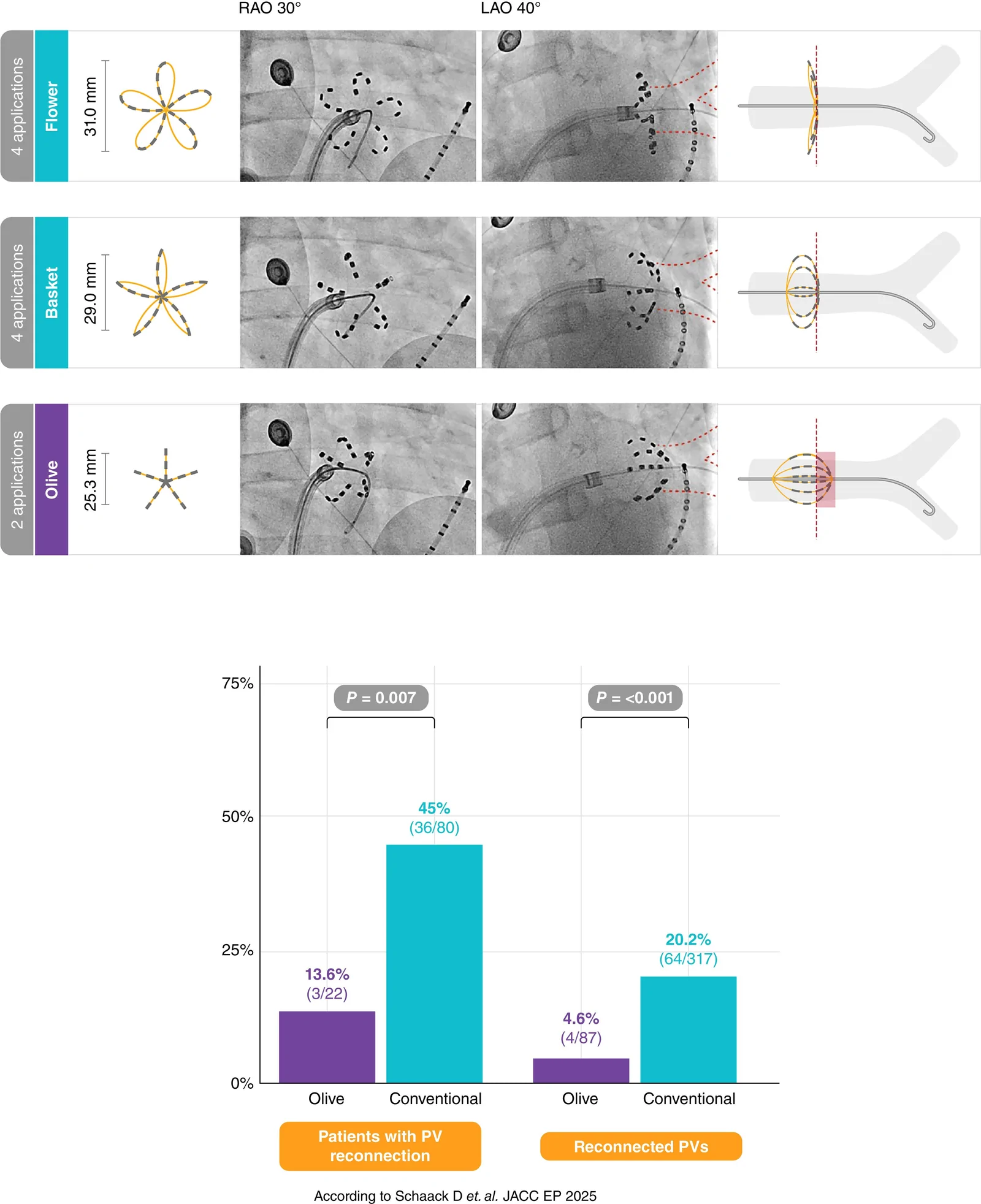
The ‘olive’ strategy workflow showing different pentaspline catheter configurations and applications. Durability data obtained from remapping studies using the ‘olive’ strategy.^23^

#### Novel PFA devices

The role of emerging PFA technologies including balloon-based systems, basket and circular catheters with or without guidewires, spherical arrays, large-footprint, and small-tip devices, as well as hybrid platforms allowing switching between RF and PFA remains to be defined and warrants evaluation in large-scale randomized and real-world studies^24–31^  *Table 2* refers to major PFA device categories and technical details.

**Table 2:** List of different commercially available PFA systems with technical details^32^

	Farapulse™	PulseSelect™	Varipulse™	Volt™	Sphere 360™	Sphere-9™	Omnypulse™	Faraflex™	STSF Dual Energy™	TactiFlex Dual Energy™	Farapoint™
Manufacturer	Boston Scientific	Medtronic	Biosense Webster	Abbott	Medtronic	Medtronic (Affera)	Biosense Webster	Boston Scientific	Biosense Webster	Abbott	Boston Scientific
Catheter architecture/strategy	Multielectrode single-shot	Multielectrode single-shot	Multielectrode single-shot	Multielectrode single-shot	Multielectrode single-shot	Focal lattice-tip dual-energy catheter	Focal PFA catheter	Focal PFA catheter	Focal dual-energy RF/PFA catheter	Focal dual-energy RF/PFA catheter	Focal PFA linear catheter
Diameter	31/35 mm	25 mm	25–35 mm	28 mm	34 mm	9 mm	12 mm	9 mm	3.5 mm	4 mm	3.5–4 mm
Catheter size	12 F	9 F shaft/9.8 F array	8.5 F	13 F	8 F	8 F	7.5 F	8 F	8 F	8 F	8 F
Over-the-wire	Yes	Yes	No	Yes	Yes	NA	NA	NA	NA	NA	NA
Steerability	NA	NA	NA	NA	NA	Bidirectional	Bidirectional	Bidirectional	Bidirectional	Bidirectional	Bidirectional
Pulse amplitude/duration	1.8–2.0 kV/µs	1.5 kV/µs	1.8 kV/µs	1.5–1.8 kV/µs	1.3–2.0 kV/µs	2 kV/µs(Pfrev: 32 A/<10 µs; EU only)	1.2–1.5 kV/µs	2 kV/µs	1.0–1.5 kV/µs	1.9–2.2 kV/µs	1.4–2.0 kV/µs
PFA Ablation mode	Bipolar	Bipolar	Bipolar	Bipolar	Monopolar	Monopolar	Bipolar	Monopolar/bipolar	Monopolar	Monopolar	Bipolar
Waveform	Biphasic	Biphasic	Biphasic	Biphasic	Biphasic	Biphasic	Biphasic	Biphasic	Biphasic	Biphasic	Biphasic
Dedicated 3D mapping	Yes	Yes	Yes	Yes	Yes	Yes	Yes	Yes	Yes	Yes	Yes

### Safety

Despite overall improvements in AF ablation, patient safety remains central in AF ablation.^33^ Due to its near non-thermal mechanism, PFA shows significantly reduced collateral injury. Large-scale data report no atrio-oesophageal fistula, no clinically relevant PV stenosis, and an extremely low risk of phrenic nerve palsy. Post-procedural haemolysis after PFA for AF treatment is believed to be common and occurs across different types of PFA platforms.^34^ Haemolysis and occasional haemodialysis (≈0.02%) have been reported, associated with insufficient catheter–tissue contact and high application numbers; specific catheter designs may mitigate this risk.^35,36^ Nevertheless, this threshold may not accurately reflect clinically meaningful haemolysis following PFA. The absence of clinical predictors or laboratory surrogates suggests that the rare risk of renal injury must be balanced with the well-established benefits of PFA when lesions are delivered in moderation while optimizing tissue contact.^37^ With growing familiarity with over-the-wire workflows, cardiac tamponade rates are reduced. Cerebrovascular events remain within expected ranges. Recently, a very low incidence (≈0.1–0.16%) of coronary vasospasm and sudden cardiac death has been reported, including delayed and coronary proximity independent cases, warranting further investigation.^38^ Please refer to chapter 5 of the PFA supplement for additional details on safety topics.

### Procedural and laboratory efficiency

The majority of novel PFA devices enable faster PVI compared to thermal ablation.^19,39^ Even if performed by operators with limited PFA experience, shorter procedure times were consistently reported.^18^ Owing to the timing of irreversible electroporation and electrical stunning, post-ablation waiting periods may be omitted without compromising acute endpoints, improving procedural efficiency and throughput. PFA seems to improve electrophysiology laboratory efficiency through short learning curves, predictable, short procedure times, and the feasibility to use deep sedation. These advantages are driven by reduced operator dependency, rapid energy delivery, simplified catheter manipulation, and the omission of post-ablation waiting periods. Consequently, this may result in more efficient laboratory scheduling, increased patient throughput, and the potential for reduced waiting times.

### Mid- and long-term efficacy

Clinical success depends on follow-up strategy, ranging from ∼60% with continuous monitoring to 70–>80% with intermittent monitoring. In early real-world practice, PFA-based PVI achieves 12-month success rates comparable to CB or RF ablation. Interestingly, AF burden assessed by loop recorders appears similar following pentaspline catheter PFA and CB PVI.^19^ Future improvements may result from enhanced PVI durability, identification of PVI non-responders, and a deeper mechanistic understanding of AF. AF burden should be considered a complementary and increasingly important endpoint, particularly in trials with continuous monitoring, but standard definitions remain necessary for comparability across studies and technologies.

### Conclusion

PFA enables PVI with short procedure times, high reproducibility, robust efficacy, and excellent safety profile. The success of PFA in PAF is primarily driven by the reliable achievement of durable PVI rather than by a fundamentally different ablation target. Further improvements in efficacy will therefore depend on identifying and addressing arrhythmogenic substrates beyond the pulmonary veins.

## PFA in persistent AF

### Pre-procedural considerations

The 2024 ESC Guidelines recommend catheter ablation for PersAF patients resistant or intolerant to antiarrhythmic drug therapy (Class I/Level A), in selected heart failure patients with reduced ejection fraction (Class IIa/Level B), or even as first-line option (Class IIb/Level C), to improve symptoms and reduce AF burden/progression in appropriately selected patients pursuing rhythm control, within a shared decision-making strategy, and a strict comorbidity optimization within AF-CARE (risk factors and stroke prevention).^40^

Although the optimal ablation strategy is still under investigation, pre-procedural planning may help to individualize lesion-set complexity (PVI-only vs. PVI+) based on PersAF duration (<6 months vs. >6 months), presence of concomitant flutter, and atrial cardiomyopathy.

Anticoagulation typically consists of uninterrupted or minimally interrupted periprocedural OAC, and long-term OAC continuation currently still follows CHA_2_DS_2_-VA recommendations irrespective of apparent rhythm outcome.^1,40^

Finally, application of PFA may be painful, and these procedures are most commonly performed under GA or deep sedation.^41^

### Procedural workflow

Evidence from PersAF cohorts shows that PFA delivers high acute PVI and procedural efficiency; however, strategy selection (PVI-only vs. PVI+) is currently still controversial and frequently individualized. Current guidelines only recommend PVI as the cornerstone of AF ablation in PersAF. In the absence of unequivocal evidence of improved efficacy, additional lesions such as posterior wall and other linear lesions or AF mapping-based focal ablation strategies are not recommended in routine clinical practice by current guidelines.^42–44^

In short-duration PersAF (<6 months), with limited substrate and PV-centric triggers, PVI-only strategy with PFA achieves rapid, wide-area lesions with high acute and durable isolation and consistent procedural efficiency. Comparative PersAF series indicate broadly similar acute success against RF or cryo, with shorter procedure times, and favourable safety profile.^43,45^

When long-standing PersAF (>6 months), extensive low-voltage zones, or non-PV drivers are present, the ablation strategy may include adding posterior wall isolation (PWI) and selected lines (roof, anterior, mitral isthmus) in some centres; however, its routine application is not recommended in current guidelines. Many data indicate feasibility of PVI + PWI using pentaspline-PFA, with encouraging 1-year outcomes that depend on lesion completeness and workflow standardization, however without proven superior efficacy.^42–45^

PFA can close gaps, complete roof/anterior lines, and target macro-re-entrant circuits. However, mitral-isthmus (MI) durability with a pentaspline PFA catheter is limited. In a long-term follow-up of a PersAF cohort, no patient had durable MI block at redo, and most recurrences were peri-mitral flutter related, often requiring coronary-sinus and/or vein-of-Marshall adjuncts to achieve final block, underscoring the need for mapping-guided lesion delivery and, where necessary, hybrid energy/epicardial approaches.^46,47^ While substantially higher lesion durability was reported following linear lesions with combined PFA and RF using a dual-energy lattice-tip catheter^2,8,48^ data on durability of PFA-only linear lesions with dedicated catheter designs and waveforms are still awaited. Importantly, given the electrical tissue ‘stunning’ caused by reversible effects of PFA, acute linear block appears to be a poor surrogate for durable transmural lesion creation and further studies are needed to establish objective criteria for durable lesion creation with PFA.^28,49^

### Safety

One of the most critical advantages of PFA is the marked reduction of classic thermal complications (atrio-oesophageal fistula, PV stenosis, and persistent phrenic palsy). Large real-world datasets and trials demonstrate low major-complication rates and support rapid adoption.^21,50^ Nonetheless, PFA-specific effects (e.g. haemolysis, coronary spasm) warrant vigilance, limit the number of applications, and protocolized monitoring, particularly when applying PFA near coronary arteries.^51,52^

In PersAF, PFA shows a favourable safety profile compared to RF and CB, with significantly less risk of durable phrenic palsy than CB, and an almost zero risk of atrioesophageal fistula, which is particularly reassuring for PWI, or for the ablation of posterior wall circuits.^21,42,44,45,50^

It is worth noting that some rare complications may only become visible with very large-scale adoption. This has been reflected in large real-world registries with the pentaspline PFA catheter such as MANIFEST-17K^50^ and MANIFEST US.^35^

### Procedural efficacy

At 1 year, freedom from atrial arrhythmia after PFA for PersAF is at least comparable to best-practice thermal strategies.^43–45^ With continuous monitoring, 12-month freedom from atrial arrhythmia recurrence was 52.0% when account for any episode ≥30 s and 71.6% when using a 0.1% burden threshold. In a randomized trial with the dual-energy lattice-tip catheter, 12-month freedom from a composite of atrial arrhythmia recurrence, use of rhythm control therapies, and acute procedural failure was 73.8%, which was non-inferior to current RF technology (65.8%).^53^ However, continuous-monitoring experience in a PersAF-dedicated programme reported sustained AF-burden reduction and lesion-durability signals, enabling more granular characterization of late recurrences than intermittent follow-up.^42^ In long-standing PersAF or markedly remodelled atria, endocardial PFA alone may be insufficient. For MI lines specifically, durability limitations with pentaspline PFA highlight a pro-arrhythmic risk of peri-mitral flutter when block is incomplete, reinforcing the role of 3D mapping, epicardial adjuncts (CS/VOM-ethanol), and/or hybrid energy to secure durable conduction block.^47^ More recently, the AVANT-GUARD randomized controlled trial compared the safety and efficacy of PVI with pentaspline catheter vs. AAD in first-line PersAF. At 1 year, the study showed that patients treated with PVI have superior freedom from recurrent atrial tachyarrhythmia and comparable safety outcomes.^54^

### 3D Mapping integration beyond PVI

Versatility and scalability across diverse patient groups are prerequisites if PFA is to become the default modality for all AF ablations; current evidence supports its application not only in PAF but also in PersAF^55^ In PersAF, substrate-directed strategies, such as PWI and broader lesion sets, are under active evaluation; early experience indicates they can be delivered safely and efficiently with PFA, although incremental clinical benefit over PVI alone remains unproven and awaits dedicated randomized trials. Beyond PVI, PFA has indeed been extended to more complex lesion architectures. In a prospective series of 215 patients (with approximately two-thirds undergoing redo procedures), pentaspline-catheter PFA achieved acute PWI in 100% of cases with a low major-complication rate (0.9%), and at redo, posterior wall durability was confirmed in 85% of patients.^56^ Despite a lower 1-year arrhythmia-free survival (53%) in that cohort, likely reflecting advanced substrate and the redo context, the demonstration of procedural safety and lesion durability in a challenging population is encouraging. More recently, a large prospective persistent-AF study employing PVI plus PWI with PFA reported favourable safety and effectiveness, further broadening the standard scope of PFA strategies.^42^ In contrast to the high durability typically seen for PVI and PWI with the pentaspline catheter, MI block and left-atrial appendage or coronary-sinus isolation are often no longer observed on 3-month remapping, highlighting limited persistence of some extra-PV targets.^46,47^ These shortcomings likely relate more to catheter geometry, originally optimized for PVI, and line orientation than to electroporation itself. As the device ecosystem expands, with varied footprints, focal tools, and adjustable or hybrid energies, treating complex PersAF substrates should become more feasible, enabling more reliable transmural lines and lesion sets.

The fact that electro-anatomical mapping (EAM) becomes pivotal once the planned lesion set extends past PVI is widely accepted. CT–EAM fusion helps define PWI boundaries and visualize the mitral isthmus; high-density voltage mapping identifies macro/micro reentrant circuits, and eventually low-voltage substrate for eventual targeted homogenization; activation mapping and differential pacing verify roof/anterior/MI block and expose epicardial breakthroughs.^42,44,45,47^ Systematic tagging of applications and documentation of validation criteria improve reproducibility and support team-wide consistency, an approach aligned with learning-curve observations from other energy platforms (*Figure 3*).

**Figure 3:**
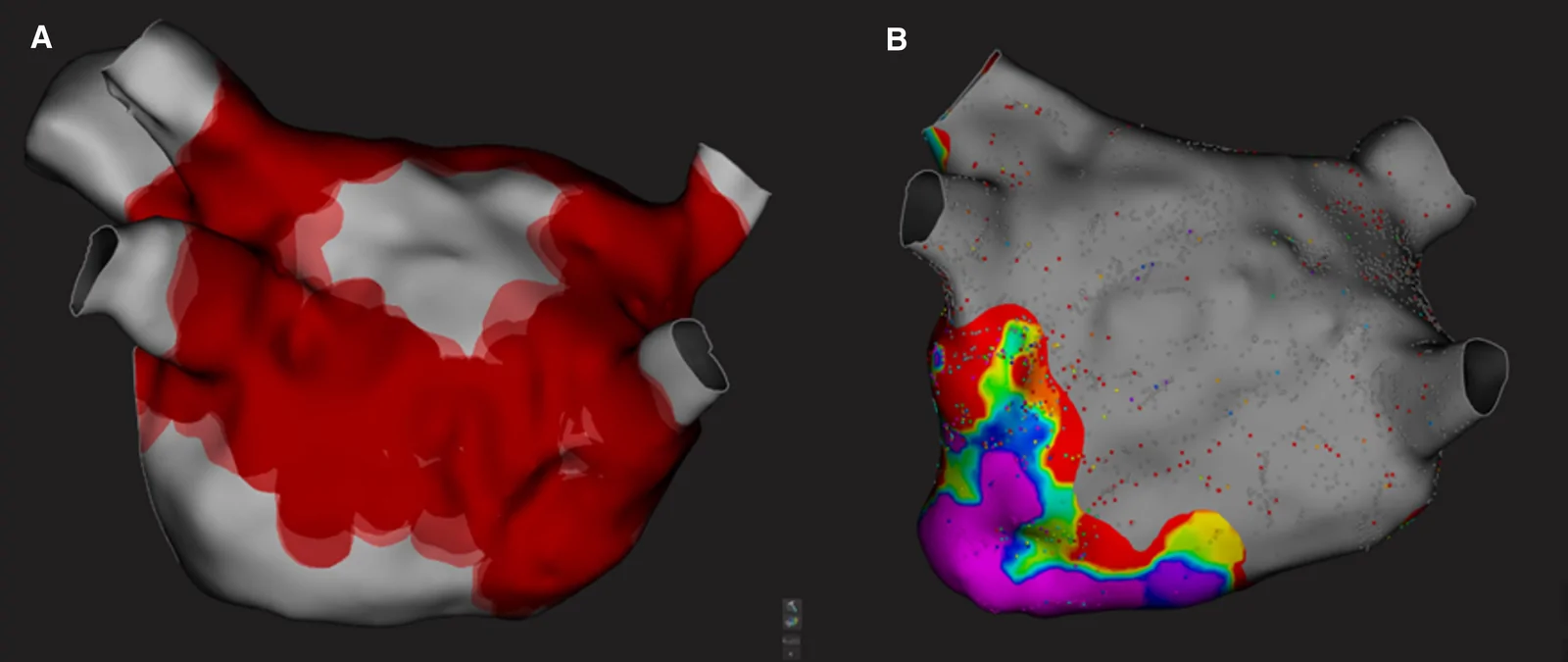
Posterior view of the left atrium in a PersAF patient after PWI performed with an Opal™ FaraNAV™ 31 mm (Boston Scientific, St Paul, MN, USA). (*A*) PFA lesion tags defining posterior wall boundaries. (*B*) Voltage map showing the posterior ‘box lesion’ type after PFA ablation.

For MI lines, EAM and eventually ICE help manage tissue thickness heterogeneity. Despite all this, adjunctive epicardial and/or vein-of-Marshall strategies should sometimes be planned upfront, when anatomy is unfavourable, or when conduction recurs and complete block cannot be obtained.^46,47^

### Upcoming studies on persistent AF ablation

Definitive randomized evidence that PVI+ substrate modification (e.g. PWI, roof/anterior lines) with PFA improves hard outcomes vs. PVI-only in PersAF is lacking. Multiple on-going randomized controlled trials are now targeting this exact question. In this context, the results of the PersAF cohort of the Beat AF Trial should bring in a near future, important insights on this crucial question.^57,58^

In addition, while PFA shows high PVI durability with the pentaspline catheter, this effect for linear lesions particularly on the MI and coronary-sinus targets remains uncertain. In this setting, evidence is still emerging on which catheter geometries, dosing paradigms, and (for hybrid energy systems) energy combinations maximize efficacy while preserving selectivity in PersAF substrate. The dual-energy lattice-tip randomized control trial showed feasibility and non-inferiority at 1 year, but longer-term PersAF-specific, head-to-head data are needed.^53^

*Table 3* summarizes currently published studies treating Persistent AF patients exclusively. Finally, real-world, multicentre registries and national programmes should continue, and future analysis will be key not only to evaluate generalizability and health system impact of PFA in PersAF ablation but also to understand learning curve, efficiency, safety signals for potentially rare events, and cost-effectiveness analysis of this energy at high scale.^59^

**Table 3:** Summary of published studies on Persistent AF

	SPHERE Per-AF (2024)	ADVANTAGE AF Phase 1 (2025)	ADVANTAGE AF Phase 2 (2025)	AVANT GUARD (2026)
Manufacturer	Medtronic	Boston Scientific _	Boston Scientific	Boston Scientific
Comparator	Contact-force RF ablation	NA	NA	Antiarrhythmic drug therapy
Study type	RCT	Single Arm	Single Arm	RCT
PFA catheter	Sphere-9 lattice-tip dual-energy catheter	FARAWAVE pentaspline catheter	FARAWAVE + FARAPOINT linear catheter	FARAWAVE pentaspline catheter
Patients	420	260	255	310
Ablation cohort	First-time ablation only	First-time ablation only	First-time ablation only	First-line rhythm-control
Ablation strategy	PVI only	PVI+ posterior wall ablation	PVI+ posterior wall ablation +CTI linear PFA (when indicated)	PVI+ posterior wall ablation
Primary Efficacy endpoint	Composite of freedom from treatment failure (arrhythmia recurrence, cardioversion, AAD initiation/escalation, or repeat ablation)	A composite of acute success of PVI and PWA; post–3-month blanking freedom from documented recurrence of AF/AFL/AT; freedom from reablation for AF/AFL/AT; freedom from any direct current cardioversion for AF/AFL/AT; and freedom from use of new or escalated doses of class I/III AADs or any post-blanking amiodarone	A composite of acute success of PVI and PWA; post–3-month blanking freedom from documented recurrence of AF/AFL/AT; freedom from reablation for AF/AFL/AT; freedom from any direct current cardioversion for AF/AFL/AT; and freedom from use of new or escalated doses of class I/III AADs or any post-blanking amiodarone	A composite of acute success and Long-term defined as freedom from the use of amiodarone through 12 months and freedom from the following after the blanking period: at least 30 s of symptomatic atrial fibrillation, flutter, or tachyarrhythmia; at least 1 h of asymptomatic atrial fibrillation, flutter, or tachyarrhythmia; electrical cardioversion or ablation for atrial fibrillation, flutter, or tachyarrhythmia; and the use of class I or III antiarrhythmic drugs (in the PFA group only)
Primary Safety Endpoint	A composite of pre-specified device-related or procedure-related serious adverse events, including death, atrio-oesophageal fistula, stroke, myocardial infarction, cardiac tamponade/perforation, PV stenosis, phrenic nerve paralysis, transient ischemic attack, thromboembolism, major vascular access complications/bleeding, heart block, gastroparesis, severe pericarditis, or new or extended hospitalization for a cardiovascular or pulmonary adverse event	Rate of predefined safety events: myocardial infarction, stroke, transient ischemic attack, thromboembolism, pulmonary oedema, unresolved phrenic nerve palsy/paresis, serious vascular access complications, heart block, or gastrointestinal disorders occurring within 7 days of the index procedure; death, cardiac tamponade/perforation, or pericarditis; or other PFA system/procedure–related cardiovascular or pulmonary adverse event within 30 days of the index procedure and PV stenosis or atrio-oesophageal fistula occurring before the 90-day follow-up	Rate of predefined safety events: myocardial infarction, stroke, transient ischemic attack, thromboembolism, pulmonary oedema, unresolved phrenic nerve palsy/paresis, serious vascular access complications, heart block, or gastrointestinal disorders occurring within 7 days of the index procedure; death, cardiac tamponade/perforation, or pericarditis; or other PFA system/procedure–related cardiovascular or pulmonary adverse event within 30 days of the index procedure and PV stenosis or atrio-oesophageal fistula occurring before the 90-day follow-up	The primary safety endpoint was device- or procedure-related serious adverse events among patients in the combined PFA group
Monitoring strategy	Continuous—implantable cardiac monitor	Continuous—implantable cardiac monitor	Continuous—implantable cardiac monitor	Continuous—implantable cardiac monitor
Mean Procedure time (min)	PFA: 101 ± 30.8 RF: 126 ± 49.2	103 ± 34.8	105 ± 36	87 ± 33
Primary efficacy	PFA: 73.8% RF: 65.8%	**63.5%**	73.4%	PFA: 56% AAD: 30%
Primary safety	PFA: 1.4% RF: 1.0%	**2.3%**	2.4%	PFA: 5.1%

## Measurable outcomes after PFA procedures

### Blanking period definition for PFA

A substantial proportion of patients exhibit early recurrences (ERs) of atrial tachyarrhythmia within the first 3 months after catheter ablation of AF. Following thermal ablation, ERs were reported in ∼30%–40% of patients under intermittent monitoring^60–65^ and 60% with ILRs.^66,67^ ERs may be promoted by a transient proarrhythmic state resulting from acute atrial injury,^68,69^ inflammation^68–70^ and autonomic nervous system alterations^71^ caused by thermal lesions. It has therefore become conventional practice to use a post-ablation blanking period during which ERs are not considered treatment failure and do not warrant reintervention. Nevertheless, numerous studies have shown that ERs are predictive of late recurrences^60,61,66,67,72–74^ and long-term AF burden.^62,66^ Crucially, the prognostic significance of ERs is strongly dependent on timing.^61–64,66^ In thermal ablation studies, 12-month freedom from late arrhythmia recurrence averaged ∼70–80% among patients without ER compared to 50–60%, 30–40%, and <10% among patients experiencing ER within the first, second, and third month post-ablation, respectively.^61–64,66^ Given these findings, the traditional 90-day blanking period was shortened to 8 weeks in the 2024 consensus statement on AF ablation.^1^

Compared to thermal ablation, PFA lesions exhibit spared vascular and nervous tissue, preserved extracellular matrix, increased apoptosis (vs. necrosis), and less inflammatory cell infiltration.^75–79^ The early post-PFA period may thus entail a milder and/or shorter proarrhythmic state. In the Single Shot Champion trial,^19^ ILR-detected ERs within 90 days occurred in substantially fewer patients randomized to PFA compared to CB ablation (38.1% vs. 58.1%, *P* < 0.001). Likewise, AF burden within the blanking period was lower in the PFA group (3.2 ± 0.8% vs. 5.5 ± 1.0%, *P* < 0.001), whereas it did not significantly differ within Days 91–365 (1.4 ± 0.4% vs. 1.9 ± 0.4%, *P* = 0.17). In ADVANTAGE AF Phase 2,^42^ ILR monitoring showed an early peak in arrhythmia burden within 30 days post-ablation, which was predictive of post-blanking healthcare utilization. In studies using intermittent rhythm monitoring, the incidence of ERs within 90 days post-PFA ranged from 6.0% to 31.6%,^80–86^ generally lower than in comparable thermal ablation cohorts,^60–64^ and ERs were independent predictors of late recurrence. To our knowledge, two studies reported monthly rates of ER after PFA with the pentaspline^83^ or a circular PFA catheter.^84^ Combining data from these two studies (631 patients, 54.7% paroxysmal AF), 90-day ERs occurred in 22% of patients. Twelve-month freedom from late recurrence was 79% among patients without ER, compared to 25% among those with ER. Patients with ER in the first, second, and third month after PFA exhibited 59%, 29%, and 9.9% freedom from late recurrence.

Some authors have suggested that the blanking period could be redefined as 1 month after PFA,^83^ or even be eliminated entirely.^87^ The blanking period does appear to be less relevant and shorter post-PFA, as ERs are less prevalent by approximately 20 percentage points compared to thermal ablation, and ERs within the second and third months post-PFA are associated with high rates of late recurrence. However, ERs in the second month may still be transient in ∼30% of patients. It therefore appears reasonable to keep using a 2-month blanking period pending further data. Adopting different endpoints for PFA vs. thermal ablation would also hinder comparisons between ablation technologies. Additional data, including detailed analysis of ILR data from recent PFA trials, may help clarify this area of uncertainty.

Finally, device-specific and monitoring-specific data are still needed before adopting PFA-specific endpoints. In our opinion, current evidence supports maintaining the consensus 8-week blanking period in clinical practice as well as for comparability purposes. However, recurrences in Month 2 (and especially Month 3) warrant particular attention (e.g. intensified monitoring) because they carry high predictive value for late recurrence.

### Durability from remapping data

Achieving durable PVI is crucial to any AF ablation strategy, even though other mechanisms are also involved in AF recurrence post-PVI, especially in persistent AF.^88,89^ In that regard, first-generation PFA systems already appear equivalent or superior to the more mature thermal ablation technologies. *Figure 4* summarizes PVI durability as reported in first-in-human studies for different commercially available and investigational PFA systems.^25,27,28,90–98^ Notwithstanding methodological heterogeneity between trials, and workflow optimization not prespecified by protocol, per-vein PVI durability with PFA reached ≥90% with most systems. For comparison, in systematic remapping studies of thermal ablation systems, including second-generation CB and contact force-sensing RF catheters, per-vein PVI durability averaged 71% (95% CI, 69–73%) with RF and 79% (95% CI, 74–83%) with CB ablation.^99^ In a recent meta-analysis^100^ comparing PFA to thermal PVI with systematic remapping data, per-vein PVI durability was significantly superior with PFA by ∼20 percentage points compared to both RF and CB ablation (*P* < 0.001). On a patient level, complete isolation of all PVs was durably achieved in 81.9% of patients with PFA compared to 54.4% with RF and 48.8% with CB ablation.

**Figure 4:**
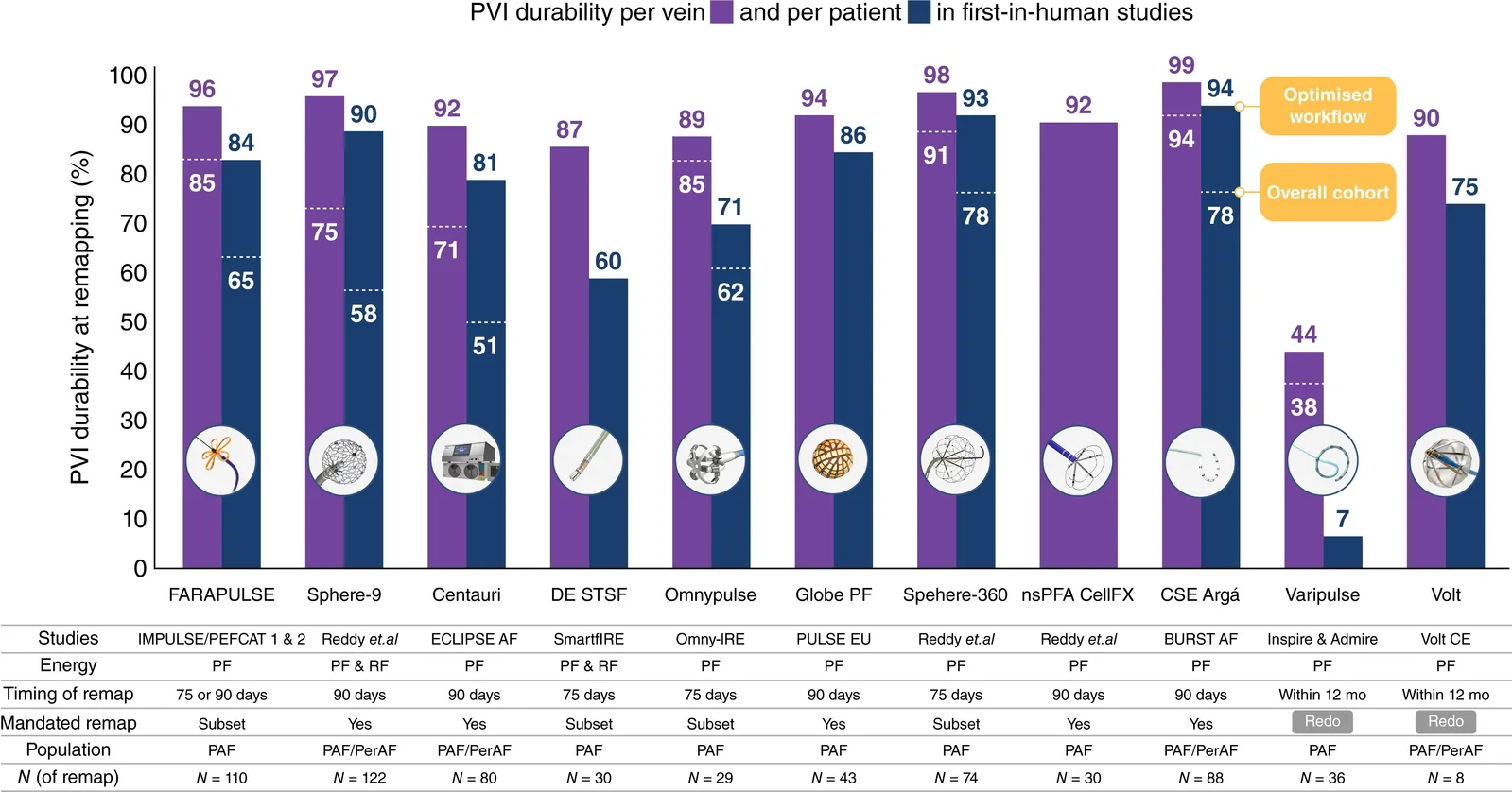
PVI durability in first-in-human studies for different PFA systems. PVI durability per vein (blue) and per patient (green; proportion of patients with all PVs durably isolated) as assessed by invasive remapping ≥75 days after index ablation. For trials with concomitant workflow optimization, solid bars refer to the optimized workflow sub-cohort (AdmIRE trial for the Varipulse studies) and dashed white lines refer to the overall cohort (pooled population if multiple studies). Per patient, PVI durability is likely more clinically relevant but was not reported in all studies. Of note, the methodology differed between trials as indicated, particularly the remapping conditions (systematic, subset, or repeat ablation only; the latter typically enriches the population in PV reconnections). Other first-in-human studies did not report PVI durability data and are not included. PAF denotes paroxysmal atrial fibrillation; PerAF, persistent atrial fibrillation; PF, pulsed field; PVI, pulmonary vein isolation; RF, radiofrequency.

Real-world data from large-scale PFA registries, including MANIFEST-REDO (*n* = 427)^17^ and EU-PORIA (*n* = 144),^101^ found substantially lower PVI durability rates averaging 71%. Nevertheless, real-world comparative data remain in favour of PFA: in a multicentre retrospective analysis of 870 propensity score-matched patients undergoing *de novo* AF ablation, per-vein PVI durability among 145 re-interventions was significantly higher following index ablation with PFA (80.9%) compared to CB (72.5%) and RF ablation (65.2%), *P* = 0.01.^102^

Data are scarce on the durability of non-PV lesions. In ADVANTAGE-AF Phase 2,^103^ durable isolation of the posterior wall was observed in 10 of 16 (62.5%) patients undergoing repeat ablation. For comparison, in the CAPLA trial,^104^ the same was achieved in 5 of 16 (31.3%) patients following contact force-enabled RF ablation. Other ablation sites, such as the mitral isthmus, may remain more challenging due to their anatomical characteristics.^105,106^ In a recent study^107^ of 236 patients undergoing AF ablation with left atrial appendage isolation, coronary sinus isolation, and mitral isthmus ablation with the pentaspline PFA catheter, acute success was 100%, 62.2%, and 100%, respectively. Systematic remapping at 3 months revealed durable isolation/block in only 4.6%, 1.3%, and 5.5%, respectively. These findings highlight the potential discrepancy between acute electrical effects of PFA and long-term results for non-PV targets. This is likely reinforcing the need for optimized catheter design. In contrast, in 122 patients undergoing systematic remapping after linear lesions with a lattice-tip dual-energy ablation catheter, durable block was achieved in 82% at the left atrial roof, 68% at the mitral isthmus, and 87% at the cavotricuspid isthmus.^28^ A small real-world study of 39 patients undergoing remapping during repeat ablation or left atrial appendage occlusion after linear ablations with the lattice-tip catheter found comparable results.^48^ Of note, both studies used various combinations of RF and PFA, making direct comparisons with other systems difficult.

Predicting durable lesion creation has been an ongoing challenge with both thermal ablation and PFA. Reversible effects of PFA (‘reversible electroporation’) may further complicate this task. Catheter-tissue contact most likely impacts PFA lesion creation, and the use of ICE to assess contact was shown to substantially improve lesion durability.^108^ Ongoing efforts including optical lesion assessment^109^ and contact sensing enabled PFA catheters^110^ may further improve durable and predictable lesion creation.

### Arrhythmia burden vs. arrhythmia recurrence

Ablation failure has been conventionally defined as atrial tachyarrhythmia recurrence ≥30 s beyond the blanking period. However, very short and sporadic arrhythmia episodes are unlikely to significantly alter the quality of life, cardiac haemodynamics, autonomic nervous system function, or cause adverse remodelling or stroke. Moreover, since many patients experience improvement in clinical status despite arrhythmia recurrences, time-to-first-recurrence analysis fails to adequately characterize response to therapy. There is now consistent evidence from both thermal ablation and PFA trials that this arbitrary endpoint is poorly correlated with meaningful clinical outcomes.^103,111,112^ Atrial arrhythmia burden, defined as the proportion of time spent in AF during a specified monitoring period and expressed as a percentage of the recording time,^20^ provides a continuous measure of rhythm status over any period of time, allowing to quantify response to therapy over time. Arrhythmia burden was shown to correlate much more closely with meaningful outcomes including AF-related symptoms, quality of life,^111,113,114^ healthcare utilization,^103,111,115^ heart failure hospitalization, new-onset heart failure,^112,116,117^ stroke,^115,118–120^ and all-cause mortality.^112,115,117^ The duration of the longest episode of uninterrupted arrhythmia is strongly associated with arrhythmia burden and also correlates with meaningful outcomes.^103,111,113,115,119,120^

The optimal thresholds to define ablation success may be specific to the clinical setting and should be relevant for outcomes. In a seminal analysis,^111^ the CIRCA-DOSE investigators found arrhythmia burden >0.1% and longest episode duration >1 h to best correlate with increased healthcare utilization (composite of emergency department visits, hospitalizations, cardioversions, and re-ablation) within 12 months after thermal PVI for paroxysmal AF. Interestingly, quality of life at 12 months was meaningfully altered only at higher thresholds of >5% arrhythmia burden and >24 h longest episode duration. These findings have been largely reproduced in PFA trials. In ADVANTAGE AF Phase 2,^103^ 12-month healthcare utilization sharply increased when ILR-based post-ablation arrhythmia burden exceeded 0.1% (RR, 11.0; 95% CI, 6.1–19.9) and when the longest episode duration exceeded 1 h (RR, 10.2; 95% CI, 5.6–18.5) (*Figure 5*). Using the traditional 30-second recurrence definition, 12-month freedom from atrial arrhythmia was 51.1%. However, 12-month effectiveness was 71.6% with ≤0.1% arrhythmia burden and 70.0% with no episode >1 h. Notably, 94% of these persistent AF patients exhibited no episode with >24 h duration post-ablation. Quality of life improved evenly across categories of residual burden and longest episode duration. Other PFA trials reported arrhythmia burden from intermittent rhythm monitoring. In the ADVENT trial,^121^ 12-month post-ablation arrhythmia burden <0.1% was associated with the lowest healthcare utilization and greatest improvement in quality of life. Residual burden <0.1% was more commonly achieved with PFA compared to thermal ablation. In the single-arm PULSED AF study evaluating a circular PFA catheter,^122^ persistent AF patients exhibited significant improvement in quality of life across all categories of residual burden, while paroxysmal AF patients experienced greater improvement with lower residual burden (<10%). This finding is consistent with ADVANTAGE AF Phase 2 and ADVENT and highlights the dependence of failure thresholds on clinical background and the relevance of quantifying response to therapy on a continuous scale.

**Figure 5:**
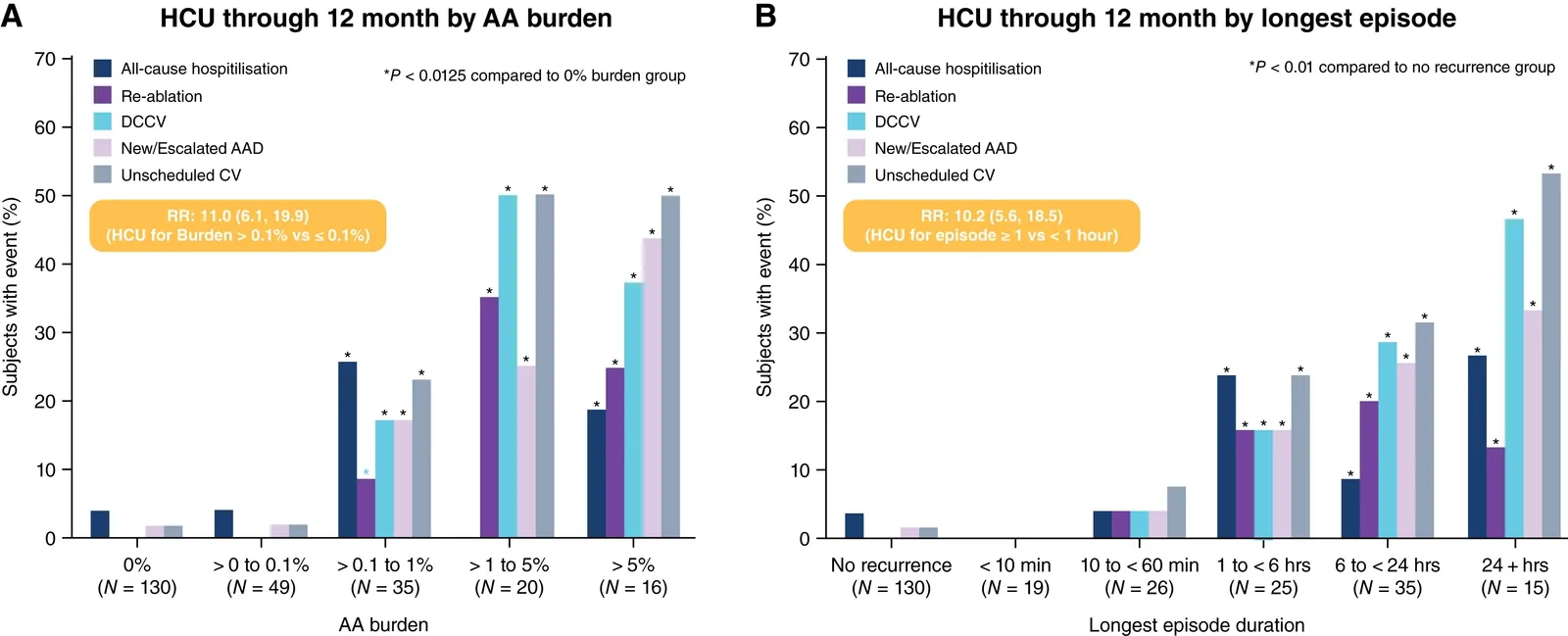
Correlation between healthcare utilization and arrhythmia burden. Healthcare utilization (HCU) as a function of atrial arrhythmia burden (*A*) and longest episode duration (*B*) assessed by implantable loop recorder 12 months after PFA for persistent AF in the ADVANTAGE AF Phase 2 trial. AA denotes atrial arrhythmia; AAD, antiarrhythmic drug; CV, cardiovascular visit; DCCV, direct current cardioversion. Reproduced with permission from Reddy et al.

Therefore, current evidence shows that arrhythmia burden is a more clinically meaningful rhythm endpoint than the traditional ≥30-second recurrence, and it is our opinion that future trials should, at least, consider burden as a complementary endpoint, particularly when continuous monitoring is used. Nevertheless, traditional definitions (based on intermittent monitoring) remain necessary for comparability across studies, technologies, and real-world data.

## Patient-reported measurable outcomes

### Introduction

Beyond procedural efficacy and safety, patient-reported measurable outcomes (PROMs) are increasingly recognized as important indicators of clinical value following AF ablation. Contemporary AF management guidelines emphasize symptom relief, functional recovery, and health-related quality of life (HRQoL) as central treatment goals, particularly where rhythm-control strategies are pursued primarily to improve patient well-being rather than survival alone.

Observational data demonstrated that PFA, through its non-thermal mechanism and relative myocardial selectivity, may have the potential to influence patient experience across multiple domains including anaesthetic requirements, procedural tolerability, post-procedural recovery, return to activity, and quality of life.^10,123–125^

### Anaesthesia, procedural tolerability, and pain management

AF ablation has traditionally been performed under either conscious/deep sedation or GA, with practice varying according to operator preference, institutional workflow, and ablation modality. Early clinical adoption of PFA frequently utilized GA, particularly during first-in-human studies and early experience with integrated and variable-loop systems. However, contemporary real-world data indicate that PFA can be successfully performed using a range of anaesthetic strategies, including GA, deep sedation, and selected conscious sedation pathways.^10,50,126^

Observational studies have demonstrated the feasibility of deep sedation protocols employing short-acting agents such as propofol, midazolam, fentanyl, and adjunctive ketamine, with high rates of acute PVI and low rates of airway intervention or conversion to GA.^50^ In one cohort, deep sedation was associated with 100% acute procedural success for PVI with no airway complications or conversions to GA.^127^ Other studies report high patient satisfaction and favourable sedation depth metrics (e.g. Richmond Agitation-Sedation Scale, visual analogue pain scores), with no requirement for invasive airway support.^128,129^ In the randomized COOPERATIVE-PFA trial, deep sedation with continuous remimazolam and ketamine achieved a lower rate of the composite of hypoxemia, hypotensive, and hypertensive events compared to both GA and propofol-opioid-based deep sedation, with similar levels of patient satisfaction.^41^

Transient skeletal muscle capture may occur during PFA energy delivery and remains an important determinant of intraprocedural comfort. The degree of myostimulation appears dependent on waveform design, pulse sequencing, catheter geometry, and energy delivery strategy. Biphasic waveforms and multielectrode circumferential catheter designs may reduce muscle stimulation compared with earlier monophasic or focal delivery systems.^125^

Conscious sedation and mild sedation protocols have also been explored, with feasibility in selected patients,^130^ though intraprocedural pain perception may be higher and comfort lower in these situations. Overall, comparative data indicate that analgesia and sedation requirements are generally higher with PFA than with thermal modalities, though sedation-related complications remain uncommon.^131^ Notably, different PFA platforms may have different sedation requirements, and focal-tip PFA catheters have been used successfully under conscious sedation.^6^

Compared with thermal ablation, PFA avoids tissue heating or freezing and may therefore reduce nociceptive stimulation related to thermal injury, oesophageal irritation, and collateral tissue inflammation. Early observational experience suggests lower post-procedural chest discomfort and less odynophagia after PFA than after RF or CB ablation.^123,124^

There remains substantial international variation in sedation pathways. In some healthcare systems, delivery of deep sedation requires dedicated anaesthetic personnel, which may influence the scalability and accessibility of PFA programmes despite favourable procedural efficiency.^10^

### Same-day discharge

Same-day discharge (SDD) following AF ablation is increasingly adopted as a patient-centred and resource-efficient care pathway. Successful SDD depends not only on procedural safety but also on patient confidence, symptom burden, early mobilization, and perceived readiness for discharge. Consensus statements support SDD in appropriately selected patients with structured follow-up pathways.^132^ While further prospective trials on SDD strategies are warranted, current meta-analysis data show that SDD after AF ablation was associated with low prevalence of post-discharge complications, re-hospitalizations/ER visits and mortality, and a similar risk compared with overnight stay.^133^

The procedural efficiency and favourable early recovery profile of PFA make it particularly suited to SDD protocols. Patients undergoing PFA frequently report minimal post-procedural chest discomfort, limited swallowing symptoms, and rapid mobilization after vascular haemostasis.^123,134^

Recent multicentre observational analyses have compared SDD rates and outcomes between PFA and thermal ablation, and suggest that SDD is more commonly achieved after PFA. One prospective study showed that 80.4% of PFA patients were discharged on the same day, compared with 57.7% after RF ablation, with lower 30-day readmission rates in the PFA cohort.^135^ Patient clinical characteristics were not significantly different between the two groups, and all procedures were undertaken under GA in both groups. The overall complication rates and use of closure devices were also not significantly different between groups, and therefore, the differences in SDD rates were postulated to be due to shorter procedural times and less perceived concern about delayed complications.

Another large comparative cohort reported higher SDD rates in PFA patients, with similar rates of major and minor complications between PFA and RF SDD groups, supporting the safety of SDD after PFA.^136^ In this study, the use of vascular closure devices was higher in the PFA group, but it was also felt that operators’ lower concern with adverse events was a major driving factor. We acknowledge, however, that these data are observational, influenced by local sedation practice, discharge pathways, patient selection, and institutional workflow.

### Post-ablation recovery and return to activity

Rapid recovery following AF ablation is a clinically meaningful patient-centred outcome. During the first post-procedural week, patients treated with PFA generally report mild and transient symptoms, most commonly related to vascular access sites rather than intrathoracic discomfort.^123,124^

Compared with historical RF cohorts, lower rates of post-procedural chest symptoms have been described following PFA, potentially reflecting reduced atrial tissue inflammation and the absence of thermal collateral injury.^124,125^ Many patients resume self-care activities within 24 h and return to light physical activity within several days, with early return to work commonly reported in observational practice^134^ (*Figure 6*).^137^

**Figure 6:**
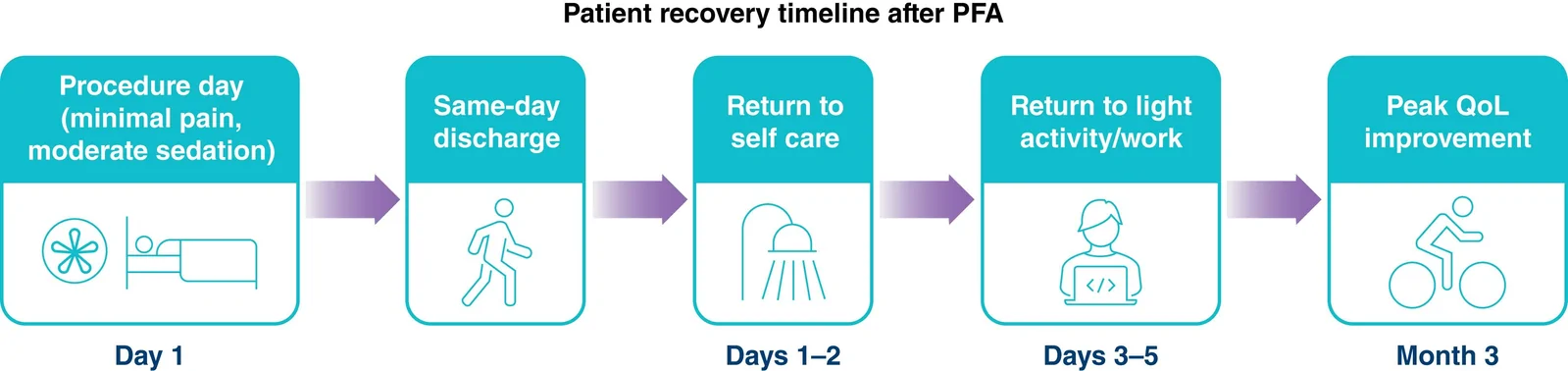
Patient recovery timeline from procedure day to return to light activity/work and peak quality of life improvement following a PFA procedure, from discharge to return to self-care and light activity/work.

Psychological recovery is increasingly recognized as an important component of PROMs after AF ablation. Although formal comparative data remain sparse, some patient experience studies suggest reassurance regarding the perceived safety profile of PFA, particularly with respect to oesophageal and phrenic nerve complications that have historically concerned patients undergoing thermal ablation.^10^

### Patient-reported outcomes and quality of life

The principal patient-centred objective of AF ablation is reduction in arrhythmia-related symptoms and improvement in quality of life. Validated AF-specific instruments such as the Atrial Fibrillation Effect on QualiTy-of-life (AFEQT) questionnaire are well established for assessing treatment benefit.

Emerging PFA trial and registry data demonstrate clinically meaningful improvements in AFEQT scores following successful ablation, including reductions in palpitations, fatigue, exercise limitation, and treatment concern. Improvements are typically evident by 3 months and may be sustained through 12 months of follow-up.^123,138^

Generic HRQoL measures, including EQ-5D domains, have also shown improvement following PFA, particularly in physical functioning and usual activities.^138^ The magnitude of benefit appears broadly consistent with that observed after successful thermal ablation, although direct comparative PROM studies remain limited, and therefore it remains uncertain whether improvements in quality of life are PFA-specific or mainly reflect successful rhythm control.

Overall patient satisfaction after PFA appears high in contemporary cohorts. Reported drivers of satisfaction include shorter procedures, rapid recovery, low symptom burden, and willingness to undergo repeat treatment if required (*Figure 7*).

**Figure 7:**
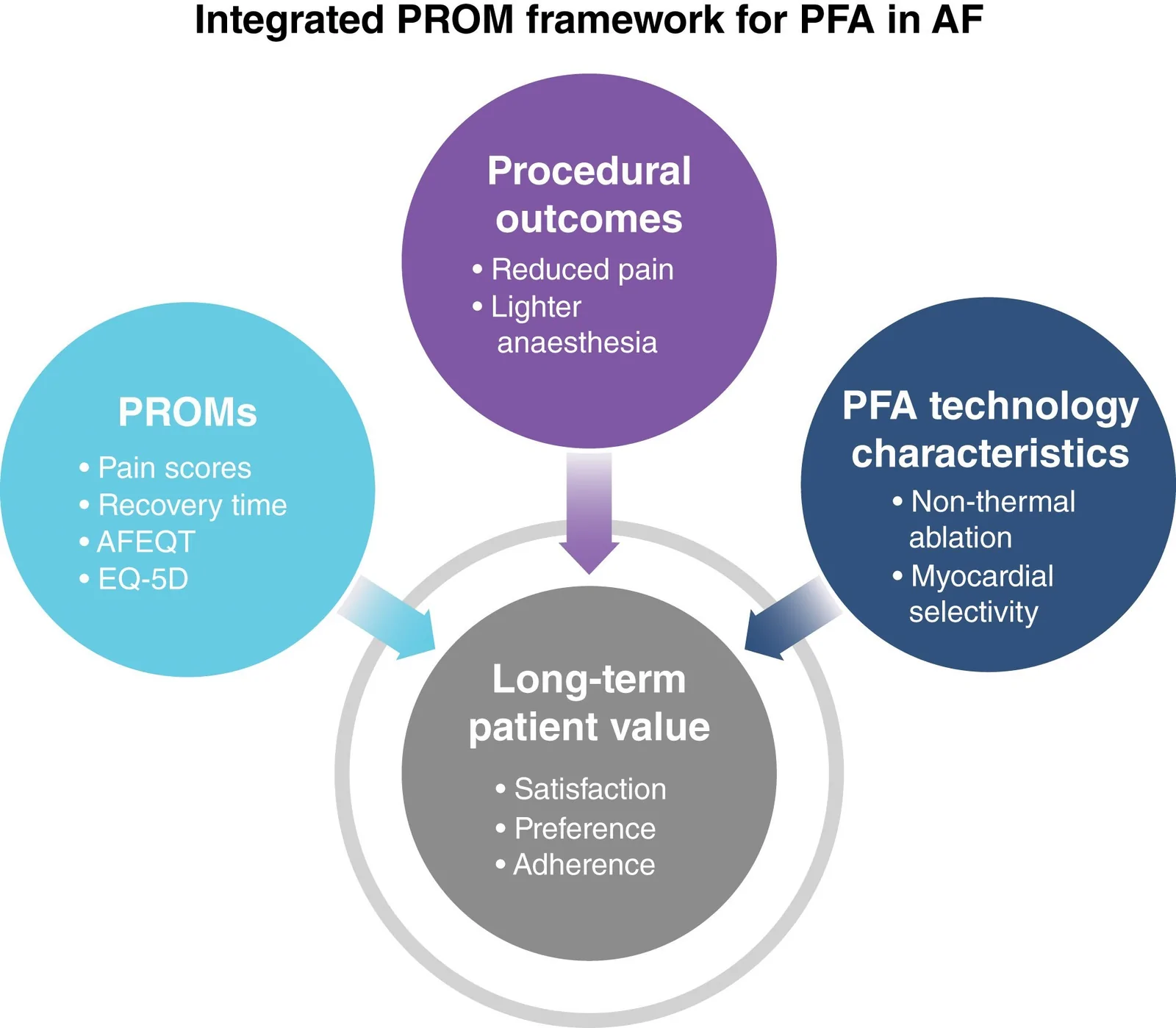
Integrated patient-reported outcome measures framework for PFA in AF leading to long-term patient value.

### Conclusions

In an era of multiple competing ablation technologies, patient experience is likely to become an increasingly important differentiator in treatment selection. Notwithstanding the observational nature of most data available, current evidence suggests that PFA offers a favourable PROM profile, with encouraging signals regarding procedural tolerability, early recovery, SDD feasibility, and post-ablation quality of life.

## Conclusion and future perspectives

PFA represents a significant advancement in the field of AF management, offering a novel, non-thermal approach that addresses many limitations of conventional ablation technologies. Across both paroxysmal and persistent AF populations, PFA has demonstrated a compelling combination of procedural efficiency, safety, and promising efficacy. Its unique mechanism of irreversible electroporation enables preferential myocardial ablation while minimizing damage to surrounding structures, translating into a more predictable safety profile and potentially reducing procedure-related complications.

In paroxysmal AF, PFA has shown high acute success rates for PVI, with encouraging mid- and long-term durability supported by remapping data. In persistent AF, where substrate complexity often necessitates more extensive ablation strategies, PFA is being explored beyond PVI, including adjunctive linear and focal lesion sets. While early results are promising, further evidence is needed to define optimal workflows and lesion strategies in this more challenging population.

Importantly, the evaluation of outcomes after PFA is evolving beyond traditional endpoints such as arrhythmia recurrence. Metrics including AF burden, lesion durability, and patient-reported outcomes—such as procedural tolerability, recovery time, and quality of life—are becoming increasingly central to assessing procedural success. The potential for SDD and faster return to normal activity underscores the broader impact of PFA on patient experience and healthcare resource utilization.

Looking ahead, continued technological refinement, integration with advanced three-dimensional mapping systems, and standardization of outcome measures will be critical. Ongoing and future clinical trials will help clarify the role of PFA in persistent AF, optimize ablation strategies, and establish long-term efficacy and safety. As evidence accumulates, PFA is poised to improve outcomes in AF ablation, with the potential to deliver more efficient, patient-centred, and durable rhythm control solutions.

## Data Availability

The data underlying this article will be shared on reasonable request to the corresponding author.
